# Evidence for common short natural *trans *sense-antisense pairing between transcripts from protein coding genes

**DOI:** 10.1186/gb-2008-9-12-r169

**Published:** 2008-12-02

**Authors:** Ping Wang, Shanye Yin, Zhenguo Zhang, Dedong Xin, Landian Hu, Xiangyin Kong, Laurence D Hurst

**Affiliations:** 1 Institute of Health Sciences, Shanghai Institutes for Biological Sciences (SIBS), Chinese Academy of Sciences (CAS) and Shanghai Jiao Tong University School of Medicine (SJTUSM), 225 South Chong Qing Road, Shanghai 200025, PR China; 2 Graduate School of the Chinese Academy of Sciences, 19A Yuquanlu, Beijing 100049, PR China; 3 State Key Laboratory of Medical Genomics, Ruijin Hospital, Shanghai Jiaotong University, 197 Rui Jin Road II, Shanghai 200025, PR China; 4 Department of Biology and Biochemistry, University of Bath, Bath, BA2 7AY, UK

## Abstract

**Background:**

There is increasing realization that regulation of genes is done partly at the RNA level by sense-antisense binding. Studies typically concentrate on the role of non-coding RNAs in regulating coding RNA. But the majority of transcripts in a cell are likely to be coding. Is it possible that coding RNA might regulate other coding RNA by short perfect sense-antisense binding? Here we compare all well-described human protein coding mRNAs against all others to identify sites 15-25 bp long that could potentially perfectly match sense-antisense.

**Results:**

From 24,968 protein coding mRNA RefSeq sequences, none failed to find at least one match in the transcriptome. By randomizations generating artificial transcripts matched for G+C content and length, we found that there are more such trans short sense-antisense pairs than expected. Several further features are consistent with functionality of some of the putative matches. First, transcripts with more potential partners have lower expression levels, and the pair density of tissue specific genes is significantly higher than that of housekeeping genes. Further, the single nucleotide polymorphism density is lower in short pairing regions than it is in flanking regions. We found no evidence that the sense-antisense pairing regions are associated with small RNAs derived from the protein coding genes.

**Conclusions:**

Our results are consistent with the possibility of common short perfect sense-antisense pairing between transcripts of protein coding genes.

## Abstract 2

A computational prediction of human coding RNA trans short sense-antisense pairs suggests that mRNA regulation by other coding transcripts might be a common occurrence.

## Background

It is now abundantly clear that RNA-RNA interactions are extremely important in the regulation of gene expression. Natural antisense transcripts (NATs) are simply RNAs containing sequences that are complementary to other endogenous RNAs [1]. They can be transcribed in *cis *from opposing DNA strands at the same genomic locus (*cis*-NATs), or in *trans *from separate loci (*trans*-NATs) [2]. Studies in several eukaryotic systems have shown that NATs can regulate gene expression at the levels of transcription, maturation, transport, stability and translation [1]. They are involved in genomic imprinting, RNA interference, alternative splicing, X-inactivation and RNA editing [3-7]. While most reports have focused on *cis *acting NATs [8-10], recently many *trans*-NATs have been discovered in human, *Arabidopsis thaliana *and other species, suggesting that antisense transcripts could be involved in complex regulatory networks in eukaryotes [11-14].

Much research has concentrated on that class of RNA whose function appears to be nothing other than regulation. Chief amongst these are microRNAs (miRNAs), a subset of *trans*-NATs that form double-stranded RNA, and subsequently induce gene silencing [15]. Hundreds of miRNAs - endogenous, approximately 22 nucleotide RNAs - have been identified in the human genome, and they repress the post-transcriptional activities of target genes through an imperfect complementary sequence, often but not exclusively in the 3'-untranslated region of the mRNA (3'-UTR) [16]. Another group of small RNAs, small interfering RNAs (21-25 nucleotides in length), are derived from long double-stranded RNAs, and they mediate the degradation of mRNAs with fully complementary sequences [17].

Most of the RNA in a human cell is likely not to be such specialist regulatory RNA. Is it likely that coding RNAs might regulate each other by sense-antisense pairing? More generally, if we are blind to whether an RNA is protein coding or not, how commonly do we see a potential sense-antisense pairing with another RNA? Here we address this issue looking for putative sense-antisense pairs by comparing all human RefSeqs with all others. Given that we know that some non-coding RNAs, especially miRNAs, are likely to pair with other transcripts [15], we restricted our analysis to protein coding mRNA. Recent studies [11-13] have identified thousands of *trans*-NATs in human mRNAs or expressed sequence tags, but they are all long *trans*-NATs. We hope to know whether it is possible that protein coding mRNA could regulate another protein coding mRNA through short sense-antisense pairing.

Given a perfect match between two mRNA transcripts, one is tempted to suppose that a sense-antisense level regulation must be happening. However, a simple null, that the perfect match is just a spurious statistical artifact, is also viable. Indeed, if one were to take a randomly generated short sequence, at some rate we would expect to find at least sometimes a perfect match somewhere in the transcriptome. How then might we know if the matches are meaningful? First, we ask whether such matches are more common than expected. To this end we employ both randomizations and alternative pairing rules. Second, we look for indications that the matches have unusual properties. Antisense regulation often involves pairing in the UTR or from transcripts originating in a UTR [13,15]. We therefore ask whether there is a per base pair enrichment for paired matches, at least one of which is a UTR. Third, we ask whether transcripts with different numbers of potential partners have different levels of expression and whether there is a difference between pairs of tissue specific genes and those of housekeeping genes. Finally, we ask whether within the coding transcript single nucleotide polymorphism (SNP) levels are lower than in flanking domains. The results suggest an unexpected richness of short sequence sense-antisense regulation between transcripts originating from protein coding genes.

## Results

### Short pairs in human transcripts

**Figure 1:**
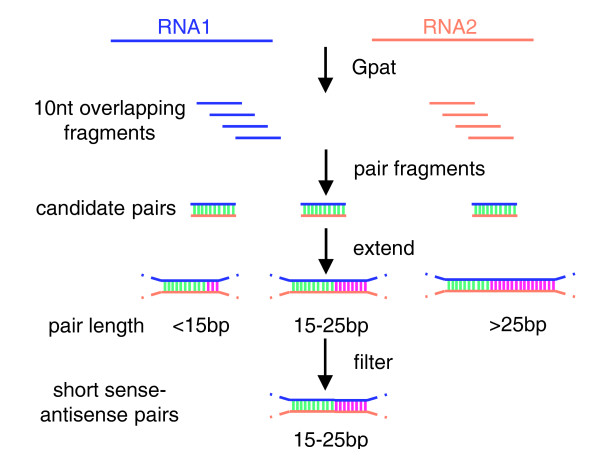
Flow chart of the search for short sense-antisense pairs.

**Figure 2:**
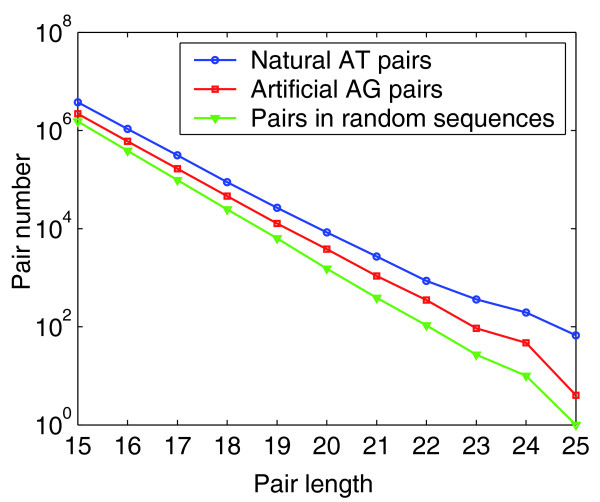
The numbers of short sense-antisense pairs. The numbers of natural pairs (AT and CG) from 24,968 human unpredicted protein coding transcripts is shown in blue. The number of artificial pairs (AG and CT) from 24,968 human unpredicted protein coding transcripts is shown in red. The number of normal pairs from a group of random sequences is shown in green.

In asking whether sense-antisense pairs are enriched in human coding mRNAs, we were cognizant that the presence of repetitive elements, either in the exons or the UTR, had the potential to lead to over-reporting of a spurious hit. We therefore first masked repetitive elements (such as interspersed repeats and sequences with a low degree of complexity) using RepeatMasker [18]. We screened (see Materials and methods; Figure 1) 5,297,874 short sense-antisense pairs of 15-25 nucleotides in 24,968 human unpredicted coding mRNA RefSeqs from release 26 (Figure 2). All 24,968 mRNAs can form short pairs with at least one other mRNA (a list of gene matches is available in Additional data file 1). Of these pairs, only 40 are in *cis *(that is, owing the gene overlap on opposite strands that are immediate neighbours and for which the transcribed domains overlap, covering the same stretch of DNA).

Is this observed rate of pairing comparable to that expected under a random null? To address this, we determined whether the pairs in human mRNA RefSeqs are markedly enriched compared to randomized sequences. Nucleotide order was shuffled using shuffleseq, provided by Emboss [19] (Figure 2). These randomized sequences had the same length and the same G+C% content as the original sample of mRNA RefSeqs. Comparing the shuffled sequences with the RefSeqs, we found that with increasing pair length, the ratio of the number of observed pairs to pairs in random sequences increased from 2.56 (with 15 bp) to 91 (with 25 bp), indicating enrichment of the RefSeqs for putative pairing domains.

To determine whether the observed enrichment is statistically significant, we compared the number of pairs in real human transcripts with that seen in 100 groups of appropriately matched random sequence. We selected 19,576 unpredicted human mRNAs from human mRNA RefSeq, and to avoid pairs redundant because of the similarity of sequence, we employed only the longest mRNA of alternative transcripts at any given locus. We selected 5,000 sequences at random from the 19,576 mRNA RefSeqs, masked repetitive sequences, and created 100 groups of random sequences using shuffleseq. We searched for short pairs in the 5,000 mRNA sequences, and in each group of random sequence. At all pair-lengths examined, the numbers of short pairs in human transcripts is far more than the maximum corresponding number of short pairs in random sequences, so short pairs are significantly rich in human transcripts (*P *<< 0.01). For example, when looking for runs of sense-antisense complementarity of minimum length 19 nucleotides, we expected to see about 250 instances of sense-antisense pairs with an upper limit of around 280; we actually observed 1,437. The same sort of enrichment is seen at all pair lengths (Figure S1 in Additional data file 2). Only 7 *cis*-pairs were found in the 5,000 genes, indicating that the pairs are almost all *trans *sense-antisense partners.

To further confirm the observed enrichment, we defined an artificial pair rule, AG and CT, and screened pairs according to this rule following the procedure described above. We found that sense-antisense pairs are more common under the natural pair rule than the artificial one (Wilcoxon sign rank test *P *< 10^-6^; Figure 2; Figure S2 in Additional data file 2). All the above results support the possibility of common mRNA-mRNA pairing or pairing between transcript fragments of mRNAs.

### Many short pairs were formed within Alu sequences of human transcripts

As noted above, to determine the statistical significance of the enrichment of short pairs in human transcripts, we masked repeat elements before we searched for possible complementary regions. However, we noticed that interspersed repeat elements with the opposite orientation, such as Alu and L1, also can form short pairs of 15-25 bp (Table 1). This is most strikingly seen within Alu, within which millions of short complementary pairs can form. Given the possibility that these Alu matches might be functionally important, we found that the non-gap complementary percentage of flanking sequences of Alu short pairs (both sense and antisense are Alu) is significantly higher than that of the non-Alu pairs (both sense and antisense are non-Alu). This means that the perfect short Alu pairs that we found can form longer imperfect pairs. For a pairing region of size x nucleotides, we define x nucleotides upstream and x nucleotides downstream of the x nucleotides pair region as the flanking region. The median non-gap pair percentage of Alu pairs in these flanking sequences (considered across all sampled values of x) is 68.75%, while that of non-Alu pairs is 25%, *P *< 2.2e-16 (Wilcoxon rank sum test; Figure S3 in Additional data file 2). Hereafter, we analyze both Alu and non-Alu pairs separately.

**Table 1:** The numbers of short pairs formed within repetitive elements

Pair length	15	16	17	18	19	20	21	22	23	24	25
ALU	2,087,406	1,511,109	983,923	886,693	653,321	628,201	543,147	419,131	277,987	200,574	178,990
L1	27,739	9,275	7,555	2,507	3,532	1,106	1,111	757	854	531	638

### The distribution of short pairs in the 5'-UTR, coding sequence and the 3'-UTR

Where in the gene does sense-antisense pairing occur? miRNAs tend to be biased to bind within the 3'-UTR of mRNA [15,16]. By contrast, it is possible that some of the complementary pairs might reflect pairing between a truncated version of an mRNA and a full length mRNA. *A priori*, such short read transcripts might be expected to be biased to 5'-UTRs and reflect premature termination of full length transcripts. Here then we ask whether, per unit base pair, pairing sites are biased as regards intra-gene position.

**Figure 3:**
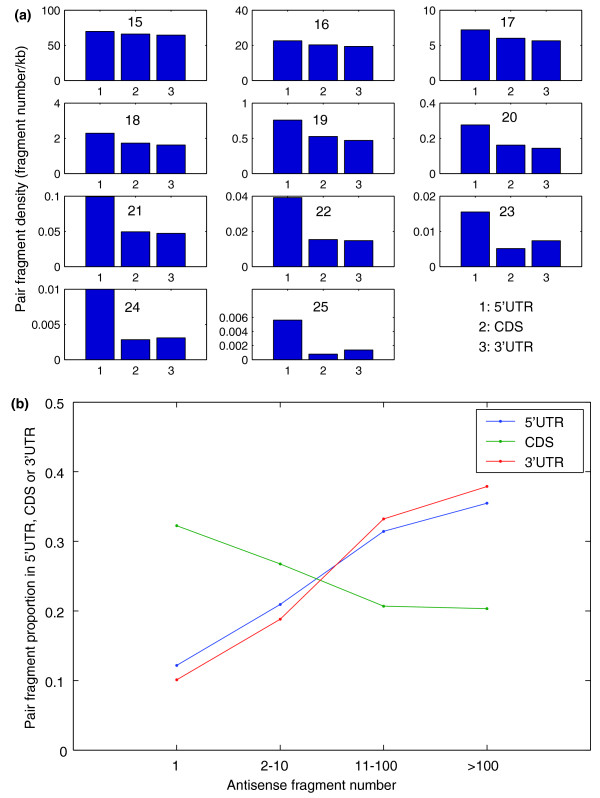
The distribution of pairs in 5'-UTRs, CDSs, and 3'-UTRs. **(a) **Non-Alu pair site density. **(b) **Distribution of Alu pair sites with different antisense site numbers.

On the basis of GenBank coding sequence (CDS) coordinate annotation, we mapped the locations of short putative sense-antisense pairs in transcripts. For each gene within which a putative sense-antisense pair is found, we determined whether the pair site was in the 5'-UTR, CDS, or the 3'-UTR of the mRNA. Examining non-Alu pairs, we found that the density of pair sites in 5'-UTRs is markedly higher than that in CDS and 3'-UTRs (Figure 3a). This is most evident for the longer pair-sites. The enrichment of putative sense-antisense pairs is not, however, due solely to putative pairing in the UTRs. If we exclude from our randomizations UTR (that is, leaving just CDS), then from shuffling the 5,000 CDSs 100 times, we still find a striking excess of putative pairs, with no randomization exceeding the observed, so CDS-CDS pairs are rich in human transcripts (*P *<< 0.01). Proportional enrichment (observed/expected) varies from around 3.6-fold at 15 bp to 7.5-fold at 22 bp.

There is a large copy number of Alu sequences in human transcripts, especially in 3'-UTRs [20], and we found that their antisense site numbers in human transcripts range from 1 to more than 100. Compared with pair sites with fewer antisense sites, those with more antisense sites (> 10) are prone to be in 5'-UTRs and 3'-UTRs (Figure 3b).

### Pair number and transcript expression values

If the putative sense-antisense pairs are functional, we might expect to see some relationship between pairing and gene expression. As pairing would typically be expected to reduce expression, we expect a gene with more putative pairs (in absolute terms) to have lower expression levels. Moreover, *a priori*, we might expect tissue specific genes to be more highly regulated than housekeeping genes, so we expect to see a difference between these two classes.

We used microarray data to analyze the relationship between pair numbers and the expression signal values of single-transcript genes in human brain and human liver. Since the expression values of different transcripts from the same gene might confuse matters, we selected 9,717 genes with only one transcript from the list of 24,968 RNAs.

**Figure 4:**
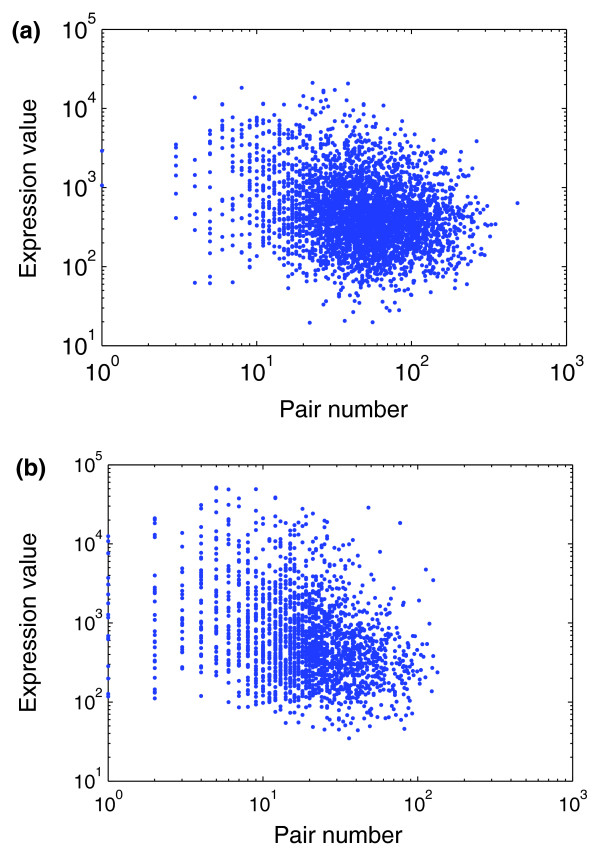
The negative relationship of pair number with gene expression; **(a) **human brain; **(b) **human liver.

We selected transcripts containing short pairing sequences from single-transcript genes that are expressed in human brain, and analyzed the relationship between absolute pair numbers and the expression signal values. We calculated how many pairs can be formed between the object RNA and other RNAs, this being the pair number of the object RNA. Considering only non-Alu pairs, 3,817 RNAs have both partners in the pair expressed in human brain. We found that the expression values and pair numbers of these RNAs are negatively correlated (*r *= -0.1928, *P *< 10^-10^, Spearman's rank correlation coefficient; Figure 4a). The same trend is seen for genes expressed in normal human liver; *r *= -0.3001, *P *< 10^-10^, Spearman's rank correlation coefficient, N = 2,065 (Figure 4b). We also found a similar relationship between pair numbers and expression signal values, if we considered both non-Alu and Alu pairs (in brain, *r *= -0.2379, *P *< 10^-10^; in liver, *r *= -0.3189, *P *< 10^-10^; Spearman's rank correlation coefficient). Generally then, the more potential partners a gene has the lower its expression level.

The above result may, however, be artifactual. While we expect expression level to be influenced by the absolute number of potential pairs, tissue specific genes are both longer and likely to be expressed at lower levels. Hence, if the sense-antisense pairs observed were meaningless artifacts, we would still expect to see a higher number of hits in lowly expressed genes. To examine this, we compared the density of pairing sites (as opposed to the absolute number) in tissue specific versus housekeeping genes.

**Figure 5:**
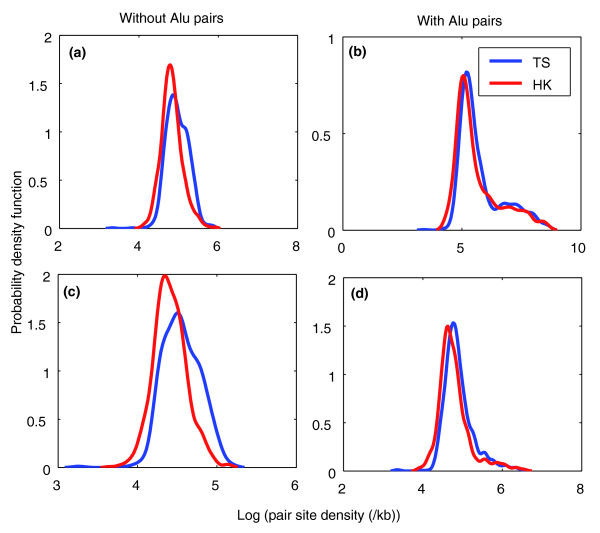
Kernel density plot showing the distribution of short pairs in tissue-specific and housekeeping genes. **(a) **Pair number density of non-Alu pairs. **(b) **Pair number density of Alu pairs. **(c) **Pair site density of non-Alu pairs. **(d) **Pair site density of Alu pairs. HK, housekeeping genes; TS, tissue-specific genes.

To this end, we derived a list of 577 tissue specific genes (expressed in only one tissue) and 659 housekeeping genes (expressed in all 79 tissues using a series of microarray data [21]). To control for gene size effects, we examined pair number density (equivalent to the number of pairing partners/bp) and pair site density (equivalent to the number of pairing sites/bp). We found that pair number density (and pair site density) of tissue specific genes is significantly larger than that of housekeeping genes (Figure 5; Wilcoxon rank sum test *P *< 10^-10^).

### SNP distribution is different in pair regions and flanking regions

If the putative sense-antisense pairing domains that we have identified are functionally relevant, then we might expect a mutation in the pairing domain to be under stronger purifying selection than one in the same genic compartment (5'-UTR, CDS, 3'-UTR) but not in the pairing domain, much as it has been reported that negative selection could be detected in exonic splicing enhancers and miRNA-binding sites by analyzing SNP distributions [22-24]. We used SNP data from dbSNP (build 127), and mapped 272,052 SNPs to mRNA RefSeqs. Because many short pairing domains are overlapping, we could not define pair regions and flanking regions according to only one pair. Instead, we selected isolated pair regions whose flanking regions could not pair with any other mRNAs in 15-25 bp. We additionally ensured that the pair region and flanking region are both in the same genic compartment (that is, the flank and pairing domain must both be in the 5'-UTR, both be in the CDS, or both be in the 3'-UTR).

**Figure 6:**
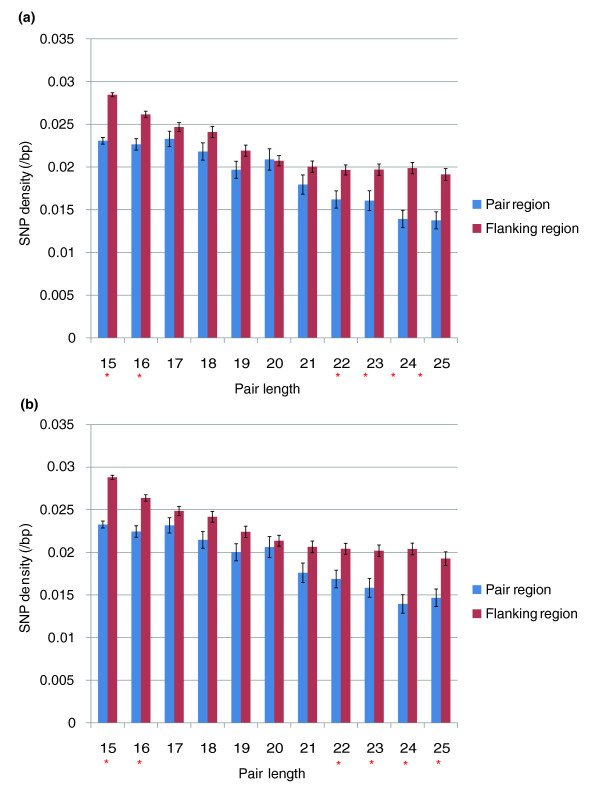
The mean SNP density difference between the pair region and the flanking region. **(a) **Short pairs without repetitive element pairs. **(b) **Short pairs with repetitive element pairs. Asterisks indicate a significant difference between the pair region and the flanking region, *P *< 0.01 (chi-square test). Error bars represent the standard error.

Excluding pairs associated with repetitive elements, we found that, overall, the SNP density in pairing regions (0.021 SNPs/kb) is significantly lower than that in flanking regions (0.025 SNPs/kb) (flanking regions are of the same length as pair regions to each side; *P *< 2.2e-16, chi-square test). Employing a more conservative paired test, we observed the same (sign rank test, *P *= 5.93e-6). With lower numbers of examples for any given pair size, it is to be expected that the 20% higher rate in flanks may be significant in some but not all instances. Indeed, we found a significant difference between flank and pair sites for domains of size 15, 16, and 22-25 bp (*P *< 0.05, chi-square test; Figure 6a), but not for sizes 17-21 bp pairs (*P *> 0.05, chi-square test). When we additionally analyzed the pairs involving repetitive elements (that is, those both with and without repetitive element involvement), the differences were significant for 15, 16, 22-25 bp pair lengths (*P *< 0.01, chi-square test; Figure 6b). When we further asked about the difference in SNP density for potential pairs both of which are expressed in the same tissue, we found these results to be robust, for both non-Alu (Figure S4A in Additional data file 2) and Alu (Figure S4B in Additional data file 2) pairs. We conclude that the distribution of SNPs is consistent with stronger purifying selection on putative short pair regions.

### Sense-antisense pairs and small RNAs

All of the above evidence is consistent with the hypothesis that sequences derived from protein coding genes mutually interact. Any putative match between two coding mRNAs need not, however, indicate that the two full-length mRNAs mutually pair. Recent evidence suggests that UTRs of coding genes can produce short non-coding RNAs [25]. Is it possible that what we have identified as domains of complementarity are really domains in which a small RNA interacts with mRNA?

To evaluate this possibility, we examined the results of an experiment to provide high-throughput sequencing of small RNAs (19-40 nucleotides) [26] and a detailed analysis of transcripts from the ENCODE region [27]. As regards the high-throughput small RNA transcriptome study, the great majority (circa 95%) of these do not derive from genic regions. Of the 1,160 that do map to transcriptional units, only 23 are identical with part of one of the mRNAs in our sample (the great majority of small RNAs derived from genic loci are located in introns). These 23 derive from 17 genes. Of these, six small RNAs completely include a putative pair region. To establish whether 6 of 23 small RNAs completely including a pair region is more than expected, we performed a simulation. As the mean length of small RNAs is 23 bp, we randomly selected 10,000 small sites of 23 nucleotides. We found that 4,533 small sites completely include a pair region; 6 of 23 is no different from 4,533 from 10,000 (*P* = 0.09, Fisher's exact test). We conclude that there is no significant difference between observed small RNAs and random small sites overlapping with sense-antisense pairs.

In the Encode study the authors fractionated small RNAs of 19-50 nucleotides in four cell lines (HelaS3, HepG2, GM006990, SK-N-SH) and used an ENCODE tiling array. Significant hybridization signals (top 1%) were termed SmRfrags (small RNA sites). To cross-check SmRfrags with our putative sense-antisense pairs, we first mapped SmRfrags to mRNA RefSeq in the ENCODE region. We found 1,514 SmRfrags that could map to mRNA RefSeq in the ENCODE region, and 55.28% of them include sense-antisense pairing domains that we found. To determine significance, we randomly selected 1,514 probes (equal to the number of SmRfrags), from a set of probes that appear not to capture small site RNAs. We found that 56.01% probes include sense-antisense pairs. There is thus no reason to suppose that our sense-antisense pairs are specifically small RNAs.

To further examine this conclusion we sorted SmRfrags according to their signal strength, and selected the top 1-100 SmRfrags, the top 101-200, and so on. The proportion matching sense-antisense pairs shows no declining tendency, consistent with a lack of correspondence between sense-antisense domains and short RNAs (Table 2). Analysis of data from all cell lines confirmed this conclusion (Table 2). We also asked whether matches in 5'-UTRs between small RNA and putative sense-antisense pairs were unusually common. To this end we selected SmRfrags that are ubiquitously expressed in all four cell lines, and then divided them according to their mapping locus into 5'-UTR, CDS, and 3'-UTR. We found no significant difference between SmRfrags and nonSmRfrags (Table 3). All of the above results suggest no concordance between small RNAs and the sense-antisense pairs that we observe.

**Table 2:** Overlap proportion of SmRfrags and sense-antisense pairs in four cell lines

Cell line	SmRfrag number	Overlap SmRfrag	Overlap nonSmRfrag	top1_100	top101_200	top201_300	top301_400	top401_500	top501_600	top601_700	top701_800
GM06990	1514	0.5528	0.5601	0.51	0.56	0.48	0.46	0.65	0.61	0.53	0.61
HelaS3	1321	0.5693	0.5678	0.51	0.52	0.56	0.56	0.63	0.74	0.57	0.52
HepG2	1175	0.554	0.5447	0.44	0.53	0.52	0.56	0.61	0.56	0.58	0.64
SK-N-SH	1225	0.5657	0.5559	0.45	0.6	0.45	0.55	0.58	0.65	0.62	0.55

**Table 3:** Overlap proportion of expressed SmRfrags in all four cell lines

	SmRfrag number	Overlap SmRfrag	Overlap nonSmRfrag	*P*-value
5'-UTR	52	0.5577	0.4038	0.116
CDS	368	0.5625	0.5109	0.16
3'-UTR	116	0.5603	0.569	0.895
Sum	536	0.5616	0.5485	0.667

The 5'-UTR, CDS and 3'-UTR are SmRfrag mapping loci. *P*-value from chi square test.

## Discussion

We have found that there are abundant possibilities for RNA-RNA interaction through short sense-antisense pairs in the human transcriptome, even if both RNAs come from protein coding genes. Is it likely that many of these short putative *trans *sense-antisense pairs have any biological function? Do they really match each other *in vivo*? Our analyses suggest that transcripts from protein coding genes do commonly functionally bind to other mRNA.

First, we found that short *trans *sense-antisense pairs exist throughout the human mRNAs at rates higher than expected under a variety of null models. Moreover, the intragenic sites where sense-antisense pairs are found are non-random. Short pairing sequences, which could form a higher number of pairs, are located preferentially at 3'-UTRs and 5'-UTRs while, by contrast, CDS regions tend to avoid high pairing sequences and favor low pairing sequences. Nonetheless, even in CDSs the rate of pairing is higher than expected under null. Pairs in UTRs may have an influence on mRNA stability, similar to miRNA [15] or *cis*-NATs [28], and translation initiation [29].

Further evidence consistent with functionality of the putative pairs comes from the finding that the SNP density in the flanking region of short sense-antisense pairing sequences is significantly (20%) higher than that in their pairing domains. Thus, these results support the suggestion that short sense-antisense pairing sequences are subject to purifying selection.

If short sense-antisense pairs are functional *in vivo*, they probably regulate gene expression or translation, like miRNA. Indeed, we do observe a correlation between pairing number of short sense-antisense pairs and the level of gene expression. Likewise, pairing density is different in tissue-specific and housekeeping genes.

While the above evidence certainly suggests that the hypothesis of common sense-antisense pairing between transcripts derived from protein coding genes is viable, it is by no means proven. For one thing, there remains a conceptual difficulty. If the pairing is long mRNA versus long mRNA, one must wonder how the tangle of folded mRNAs can actually come to pair. Indeed, it may be no accident that miRNAs are micro, as this must ease the pairing with the sense transcripts and prevent the miRNA finding highly convoluted and difficult to unravel secondary structure (they typically have one hairpin structure). One possible resolution of this quandary is the finding of an excess of pairs with one partner in 5'-UTRs. This suggests the possibility that premature termination of transcription of a full length mRNA could produce a regulatory RNA that is dominantly or exclusively derived from the 5'-UTR and that could possibly function like a miRNA. This would be compatible with recent evidence suggesting that UTRs of coding genes can produce short non-coding RNAs [25] and that the rate of transcription initiation is much higher than the rate of elongation resulting in full length transcripts [30]. However, from the above examination of small RNAs, we conclude that either this does not explain our data or that the two analyses failed to identify the relevant transcripts. We are left to conclude that, assuming the indications we have found for sense-antisense pairing between mRNAs are real, mRNA-mRNA pairing is a possible model of gene regulation. Confirmation of this will require experimental validation.

## Conclusion

We found that short *trans *sense-antisense pairs between human mRNAs are more common than expected by chance. A reduced SNP density in pairing domains and correlations with expression parameters suggest that this pairing could be functionally important. We found no evidence that mRNA-mRNA short pairs are due to pairing involving small RNAs. By exclusion, we propose that mRNA-mRNA pairing may be functionally important.

## Materials and methods

### Searching for short sense-antisense pairs in transcripts

To find all 15-25 bp short pairs in human mRNAs, we used the GNU Pattern (Gpat) software [31], which is an open source implementation of the Splash algorithm [32] for pattern discovery and developed in our laboratory, and screened out all 10 nucleotide-length overlapping fragments (N→N+9, N+1→N+10, ...) across mRNA RefSeqs (Figure 1). Next, for each fragment F, we found its reverse and complement fragment F', and paired these two fragments into a group to be a candidate pair *P*. To avoid redundant 15-25 bp short pairs in the result, we extended each candidate pair *P *in a single direction, only searching the next nucleotides in the 5' of F and 3' of F', and found over what nucleotide length they matched perfectly. We then identified all pairs with a matched length of 15-25 bp. Because polyadenine is a specific structure at the tail of mRNA, and could form plenty of pairs with other RNA polythymines, we defined more than three adenines at the tail of mRNA simply as polyadenine, and excluded the pairs in these regions. For the same reason, we excluded pairs with a repeat region in RNAs determined with RepeatMasker.

### Calculation of pair site density and antisense site number

To determine pair site density, we determined how many pair sites there were in 5'-UTRs, CDSs and '-UTRs, and then divided the transcript length of 5'-UTRs, CDSs and 3'-UTRs by these numbers, excluding repetitive regions. For each pair site, we found the corresponding antisense site number. For a given mRNA, there are many short sites that can pair with other mRNAs. For each pair site we can determine how many pair partners it has (this being the antisense site number of this pair site), and whether it is in the 5'-UTR, CDS or the 3'-UTR of the focal gene. As the majority of pair sites have only one antisense site, we divided them into the following four groups: 1, 2-10, 11-100, and > 100. Then, we calculated the proportion of each group in 5'-UTRs, CDSs and 3'-UTRs of the focal mRNA.

### Gene expression values from a series of microarrays

We collected gene expression data of human normal brain and liver (two samples of each tissue) from a series of published microarray experiments [21] based on the Affymetrix U133A platform. Arrays of each tissue were analyzed in the same manner. We considered the mean of the signal values of two samples of the same tissue as the signal value of each probe. The mean value of duplicated probes, that is, probes representing the same gene, was calculated, and if more than half of duplicated probes were present, we defined the gene transcript as present, otherwise absent. As U133A could not discriminate between alternative transcripts, we selected the genes having only one transcript from the list of 24,968 RNAs to determine the relationship between pair number and expression value.

We used data from a series of published microarray experiments [21], including 79 human normal tissues. For two samples of the same tissue, we defined the probe present if the expression was found for at least one. If more than half of duplicated probes for one gene were present, we defined the gene present, otherwise absent. We reserved those pairs in which the two genes are expressed in at least one tissue for SNP density analysis.

### Searching for sites including both pair region and flanking region

To assess SNP density in pairing sites and flanking sites, we mapped all short 15-25 bp non-Alu pairing domains to mRNAs, and then divided every mRNA into 5'-UTR, CDS and 3'-UTR. The SNP density in pairing sites is easy to define. To define non-pairing sites, we considered sites non-pairing at all pairing lengths. For example, for 15 bp, we found short sites including a pair region and flanking region in each genic compartment (5'-UTR, CDS, 3'-UTR). The pair region is pairing with another mRNA at 15 bp, and the flanking region is without any pair at 15-25 bp. Then we calculated the SNP density of the pair region and the SNP density of the flanking region, and compared the density of the pair region and the flanking region using a chi-square test. For these instances involving Alu pairs, we mapped all short 15-25 bp with the Alu pair domain to mRNAs, and followed the same procedure to compare the SNP density difference between the pair region and the flanking region.

## Abbreviations

CDS: coding sequence; miRNA: microRNA; NAT: natural antisense transcript; SNP: single nucleotide polymorphism; UTR: untranslated region.

## Authors' contributions

XK, LH, PW and LH conceived and designed the experiments. PW, SY, ZZ and DX performed research. LH, XK, PW and LH analyzed the data. PW, LH and XK wrote the paper. All the authors read and approved the final manuscript.

## Additional data files

The following additional data are available with the online version of this paper. Additional file 1 provides gene pairs that show mutual sense-antisense pairing. Additional file 2 includes supplementary Figures S1-S4. Figure S1: distribution of the pair numbers in 100 groups of 5,000 random sequences (bar). The pair number of 5,000 randomly selected human transcripts is indicated with an asterisk. Figure S2: kernel density distribution of natural AT and CG pairs (blue) and artificial AG and CT pairs (red) of mRNA. Figure S3: non-gap pair percentage of flanking sequences of short 22 bp pairs; (a) non-Alu pairs; (b) Alu pairs. Figure S4: mean SNP density difference between the pair region and the flanking region; (a) short pairs without repetitive element pairs and both expressed in at least one tissue; (b) short pairs with repetitive element pairs and both expressed in at least one tissue. Asterisks indicate a significant difference between the pair region and the flanking region, *p *< 0.01 (Wilcoxon sign-rank test).

## Supplementary Material

Additional data file 1 Gene pairs that show mutual sense-antisense pairing. Click here for file

Additional data file 2 Figure S1: distribution of the pair numbers in 100 groups of 5,000 random sequences (bar). The pair number of 5,000 randomly selected human transcripts is indicated with an asterisk. Figure S2: kernel density distribution of natural AT and CG pairs (blue) and artificial AG and CT pairs (red) of mRNA. Figure S3: non-gap pair percentage of flanking sequences of short 22 bp pairs; (a) non-Alu pairs; (b) Alu pairs. Figure S4: mean SNP density difference between the pair region and the flanking region; (a) short pairs without repetitive element pairs and both expressed in at least one tissue; (b) short pairs with repetitive element pairs and both expressed in at least one tissue. Asterisks indicate a significant difference between the pair region and the flanking region, *p *< 0.01 (Wilcoxon sign-rank test). Click here for file
