# Supplementary material for: Evidence for common short natural trans sense-antisense pairing between transcripts from protein coding genes
Source: Genome Biol. 2008 Dec 2;9(12):R169. doi: 10.1186/gb-2008-9-12-r169 (PMC2646273; doi:10.1186/gb-2008-9-12-r169)

Figure S1: Distribution of the pair numbers in 100 groups of 5,000 random sequences (bar). The pair number of 5,000 randomly selected human transcripts (*).

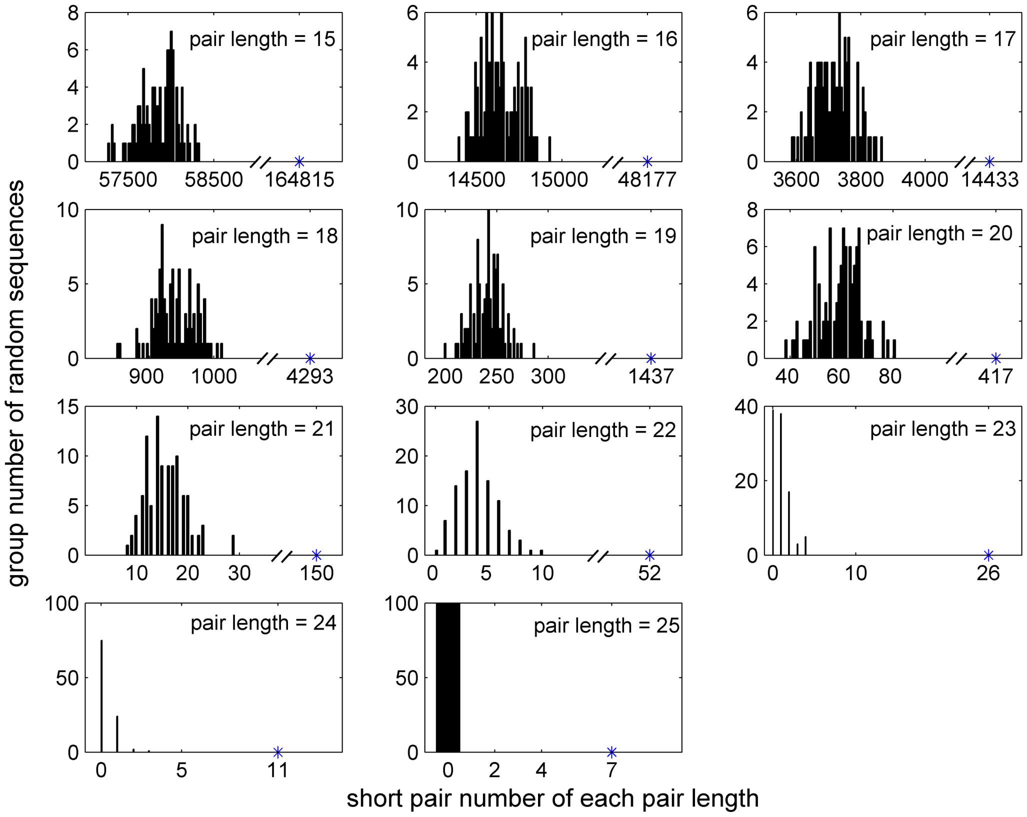

Figure S2: The kernel density distribution of natural AT and CG pairs (blue) and artificial AG and CT pairs (red) of mRNA.

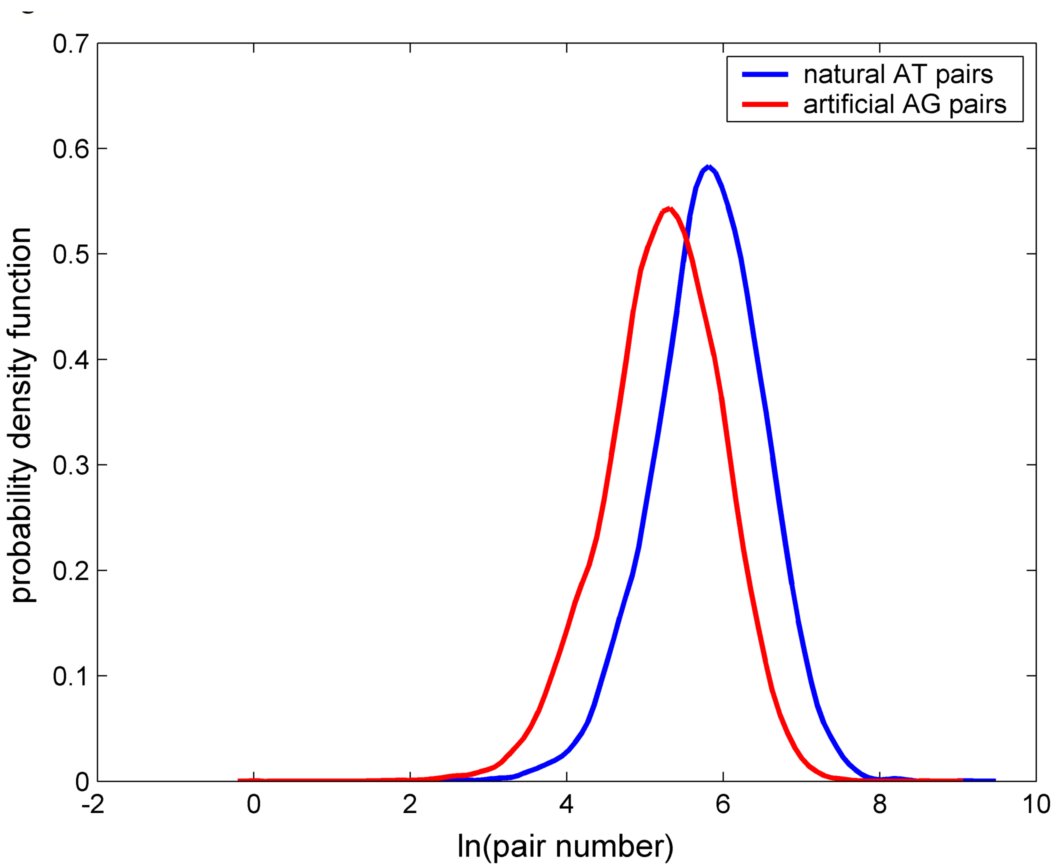

Figure S3: The non-gap pair percentage of flanking sequences of short 22bp pairs. (A) non-Alu pairs; and (B) Alu pairs.

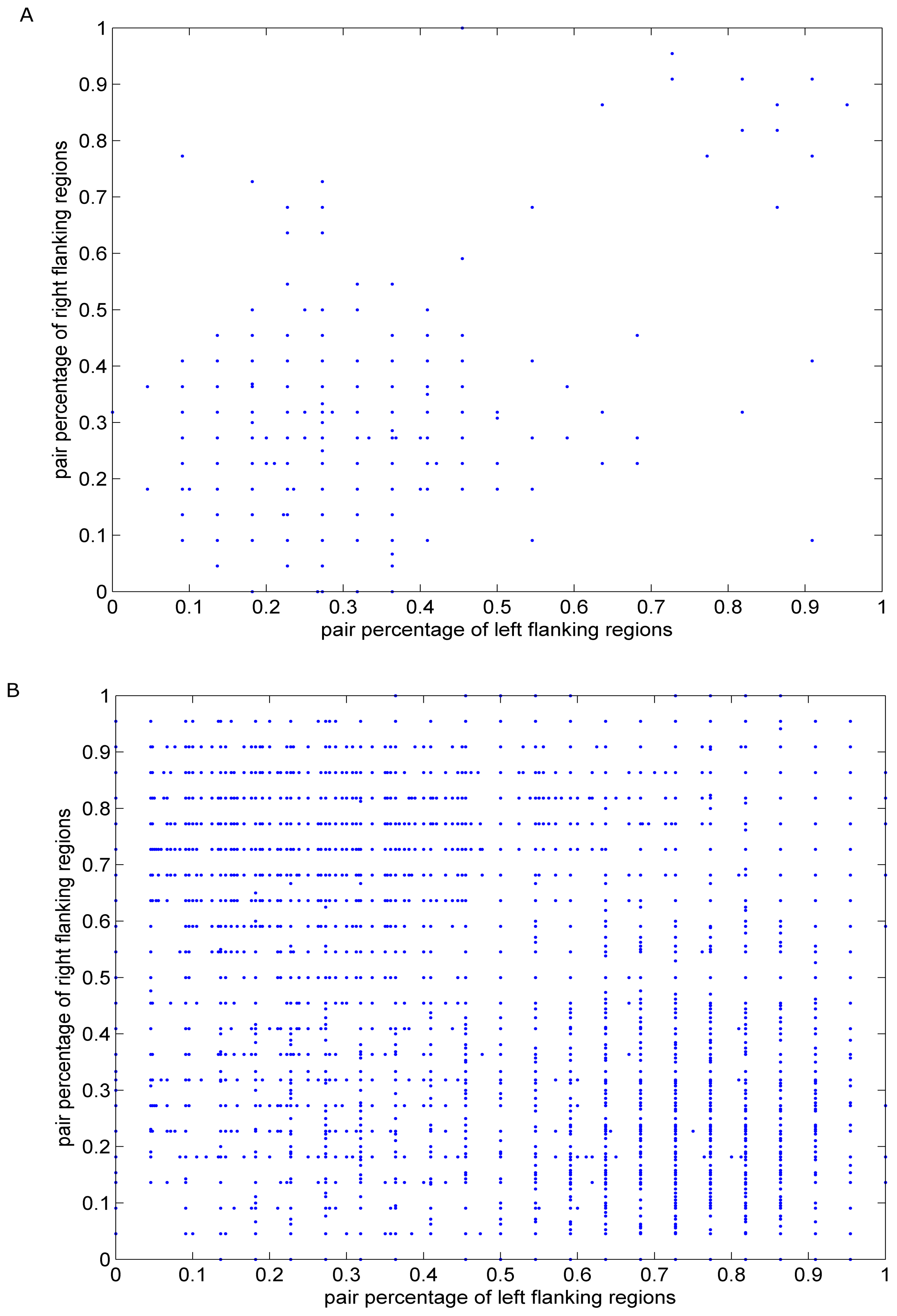

Figure S4: The mean SNP density difference between the pair region and the flanking region. (A) Short pairs without repetitive element pairs and both expressed in at least one tissue. (B) Short pairs with repetitive element pairs and both expressed in at least one tissue. *, Significant difference between pair region and flanking region, p <0.01 (wilcoxon sign-rank test).

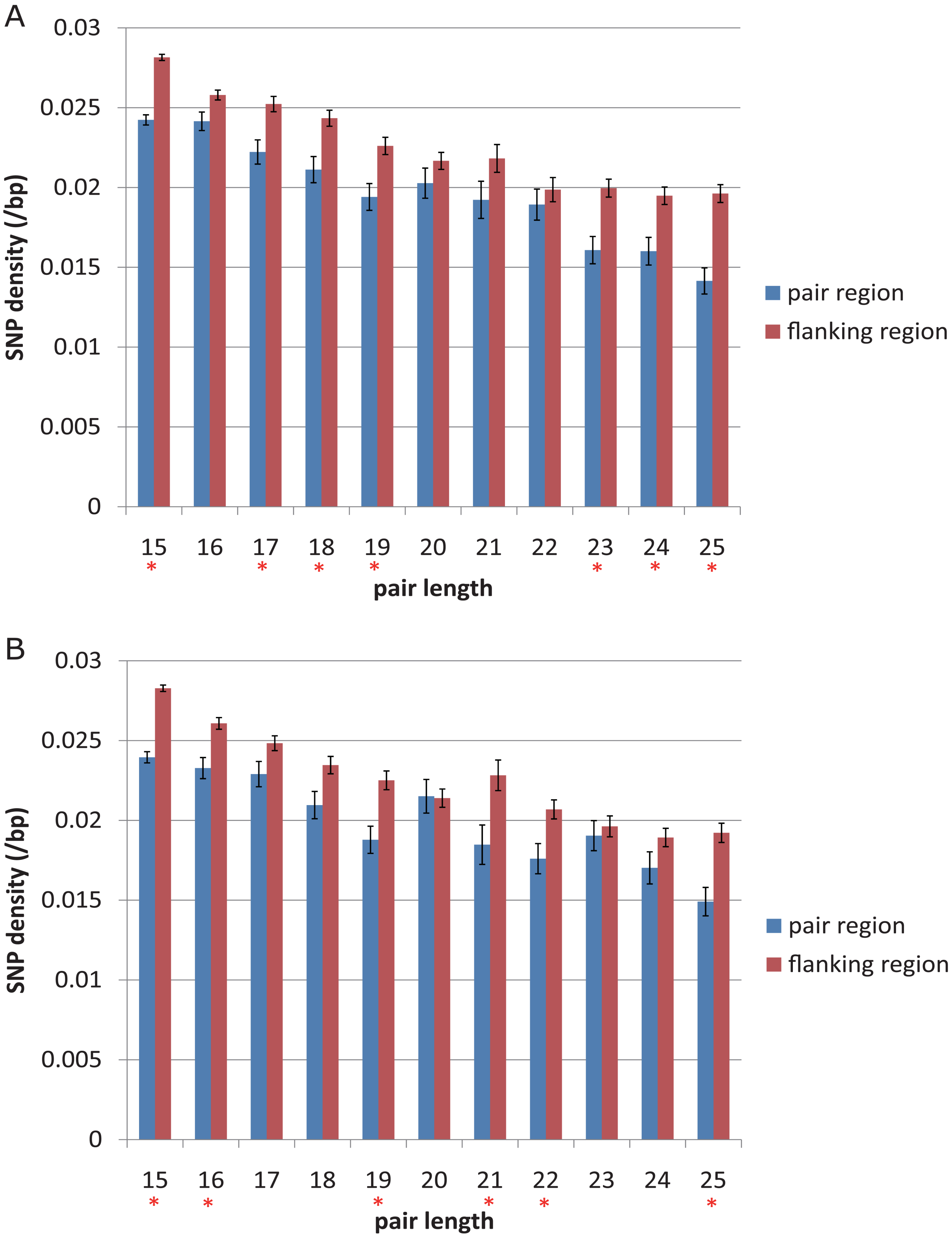

Supplement: Additional data file 2 — Figure S1: distribution of the pair numbers in 100 groups of 5,000 random sequences (bar). The pair number of 5,000 randomly selected human transcripts is indicated with an asterisk. Figure S2: kernel density distribution of natural AT and CG pairs (blue) and artificial AG and CT pairs (red) of mRNA. Figure S3: non-gap pair percentage of flanking sequences of short 22 bp pairs; (a) non-Alu pairs; (b) Alu pairs. Figure S4: mean SNP density difference between the pair region and the flanking region; (a) short pairs without repetitive element pairs and both expressed in at least one tissue; (b) short pairs with repetitive element pairs and both expressed in at least one tissue. Asterisks indicate a significant difference between the pair region and the flanking region, p < 0.01 (Wilcoxon sign-rank test). [file gb-2008-9-12-r169-S2.pdf]
